# Increased insulin resistance compounded by reduced insulin sensitivity drives the "Fat Aussie" (*Alms1foz/foz*) model of Alström syndrome towards obesity and type 2 diabetes mellitus

**DOI:** 10.1186/2046-2530-1-S1-P86

**Published:** 2012-11-16

**Authors:** D Girard, N Petrovsky

**Affiliations:** 1 Flinders University, Adelaide, Australia

## Background

The Fat Aussie mouse carries a spontaneous mutation (*foz*) resulting in a premature stop codon in exon 8 of the *Alms1* gene and is a model for Alström syndrome. From 60 days of age onwards *Alms1foz/foz* mice exhibit a strong metabolic phenotype leading to severe obesity and type 2 diabetes mellitus (T2DM).

## Objective

Investigate whether peripheral insulin resistance or a beta-cell insulin secretory defect comes first in young, non-obese pre-diabetic *Alms1foz/foz* mice.

## Methods

Insulin tolerance tests (ITT), glucose tolerance tests (GTT), fasting and post-challenge serum insulin levels and HOMA-IR score determination were performed in age and sex-matched young lean *Alms1foz/foz* mice and wildtype littermates.

## Results

When compared to wildtype mice, young *Alms1foz/foz* mice had a significantly reduced response to insulin during ITT while no differences were observed in glucose and endogenous insulin levels during GTT. Male but not female *Alms1foz/foz* mice had significantly higher fasting hyperinsulinemia and HOMA-IR scores compared to wildtype littermates.

## Conclusions

These data indicate that insulin resistance precedes obesity in young *Alms1foz/foz* mice at a time that beta-cell function isn’t affected. This suggests that early peripheral insulin resistance is an inherent primary consequence of the *Alms1foz/foz* mutation and may thereby drive the subsequent metabolic complications in this model.

